# Domestication of a Wild *Polyporus tuberaster* and Antioxidant Activity of Its Polysaccharide Extracts

**DOI:** 10.3390/jof12030196

**Published:** 2026-03-09

**Authors:** Jiarong Cai, Huijuan Sun, Lei Gao, Rongmei Huang, Xin Hu, Junsheng Fu

**Affiliations:** 1 College of Life Sciences, Fujian Agriculture and Forestry University, Fuzhou 350002, China; cjr202501@163.com (J.C.); hx1962024@163.com (X.H.); 2 Institute of Vegetables, Xizang Academy of Agricultural and Animal Husbandry Sciences, Lhasa 850000, China; m18898002012@163.com (H.S.); gaoleilei3@163.com (L.G.); 3 Xizang Autonomous Region Edible Fungi Resources and Application Technology Innovation Center, Lhasa 850000, China; 4 Fujian Edible Fungi Technology Promotion Station, Fuzhou 350000, China; 15280413178@163.com

**Keywords:** *Polyporus tuberaster*, isolation and identification, biological characteristics, polysaccharides, antioxidant activity

## Abstract

To exploit wild mushroom resources in Tibet, a wild strain from Tibet was isolated, identified as *Polyporust tuberaster*, and domesticated for fruiting. Its growth characteristics were studied, and the antioxidant activities of the intracellular polysaccharides extracts (IPS) extracted from the mycelium and the extracellular polysaccharides extracts (EPS) from the fermentation broth were compared. The optimum carbon source for mycelial growth is fructose; the optimum nitrogen source is ammonium sulfate; the optimum pH is 5; and the optimum temperature is 20 °C. Both extracellular polysaccharide extracts (EPS) and Intracellular polysaccharide extracts (IPS) exhibited antioxidant capacity. The *IC*_50_ values of EPS for scavenging OH·, ABTS·^+^, and DPPH· were 1.357, 0.125, and 0.683 mg/mL, respectively, while those of IPS were 0.595, 0.152, and 3.401 mg/mL. At 5 mg/mL, the FRAP values were 0.1582 (EPS) and 0.1708 (IPS). In cultivation, mycelium fully colonized bags after 32 d at 23 °C in darkness. Primordia formed within 12 d under 95% humidity with scattered light, and mature fruiting bodies developed after 24 d at 85–90% humidity and 20–23 °C, yielding an average fresh weight of 41.27 g per bag for the first flush. This study provides a basis for further development of *P. tuberaster*.

## 1. Introduction

*Polyporus tuberaster* (Jacq.) Fr. belongs to the kingdom Fungi, phylum *Basidiomycota*, class *Agaricomycetes*, order *Polyporales*, and family *Polyporaceae* [1,2]. Its fruiting body is annual. The pileus is circular, semicircular, or fan-shaped, with a yellowish/brown to ochre surface marked by tea-brown patches, thinning from the base toward the margin. The pores are pale yellowish/brown to tea-brown, and the context is white to creamy, fleshy to leathery. The stipe is lateral and covered with fine hairs [3].

The *Polyporaceae* family, as an important group of macrofungi, possesses not only a strong lignin-degrading capacity [4] (e.g., laccase, manganese peroxidase) but also a rich variety of metabolites (such as polysaccharides [5,6], triterpenoids [7], phenolics [8], and sterols [9]), making it a research focus for bioenergy development, environmental pollution remediation, and drug discovery. However, there are significant functional differences among species within *Polyporaceae*. For instance, *Ganoderma* spp. are renowned for the immunomodulatory activities of their polysaccharides and triterpenoids [10], while peroxidases from *Trametes hirsuta* are widely used in dye detoxification [11]. As a member of the *Polyporaceae*, *P*. *tuberaster* also holds considerable economic value in wastewater treatment, biofilm applications, and papermaking [12]. Studies have shown that this fungus has a strong ability to produce laccase [13], can generate aromatic compounds with high sensory evaluation scores, making it suitable for beverage development [14]. Furthermore, its fermentation products exhibit inhibitory effects on various cancer cells and, compared with traditional anticancer drugs, have the advantage of not increasing the proportion of side population cells [15]. Clearly, *P. tuberaster* possesses significant development potential.

Although *P. tuberaster* sclerotia are widely distributed, research on their metabolic and functional characteristics remains very limited. Existing studies have shown that microorganisms inhabiting extreme environments, such as high altitudes, cold temperatures, and intense ultraviolet radiation, often evolve unique physiological and metabolic adaptations [16,17,18,19]. Therefore, the *P. tuberaster* sclerotia strain isolated from the Qinghai–Tibet Plateau may possess distinctive metabolic traits and ecological functions due to long-term adaptive evolution in extreme environments, which endows it with significant scientific exploration value. However, current studies on *P. tuberaster* have focused mainly on taxonomy and resource surveys [20], with little reported on its biological characteristics and domestication for cultivation, especially for strains collected from plateau regions. Therefore, this study takes a *P. tuberaster* strain isolated from the Nyingchi City region of Tibet as the research subject, systematically analyzes the biological characteristics of its mycelium, evaluates the antioxidant capacity of intracellular and extracellular polysaccharide extracts from the mycelium, and establishes domestication and cultivation techniques. The aim is to provide a theoretical basis for the conservation of this high-potential species, the production and development of its bioactive compounds, and the development of a distinctive plateau fungal industry.

## 2. Materials and Methods

### 2.1. Experimental Materials

The fruiting bodies, collected from Yigong Township, Bomê County, Nyingchi City, Tibet Autonomous Region, were wild specimens with the collection number X21196. The mycelia were obtained using the tissue isolation method. Fresh fruiting bodies were selected, and their surfaces were disinfected with 75% ethanol. Under sterile conditions in a laminar flow cabinet, internal tissue blocks (approximately 2–3 mm^3^) were aseptically cut from the junction of the cap and stipe, inoculated onto PDA medium, and cultured in the dark at 25 °C for 5–7 days. After the new mycelia grew around the tissue blocks, the hyphal tips were transferred to fresh PDA slants for purification culture. The purified strain was deposited at the Mycological Research Center of Fujian Agriculture and Forestry University.

### 2.2. Chemical Reagents and Instruments

Potassium persulfate, H_2_O_2_ (30%), maltose, mannose, fructose, soluble starch, sucrose, glucose, yeast extract, beef extract, vitamin C (Vc), magnesium sulfate, potassium dihydrogen phosphate, Vitamin B1 (VB_1_), hydrochloric acid, anhydrous ethanol, sodium hydroxide, salicylic acid, urea, and other reagents (analytical grade, purchased from China National Pharmaceutical Group Corporation, Beijing, China), 2,2′-Azino-bis(3-ethylbenzothiazoline-6-sulfonic acid) diammonium salt (ABTS), 2,4,6-Tris(2-pyridyl)-s-triazine (TPTZ), 2,2-Diphenyl-1-picrylhydrazyl (DPPH). (Shanghai Yuanye Bio-Technology Co., Ltd., Shanghai, China). Molecular Devices SpectraMax i3 multifunctional microplate reader (Molecular Devices, LLC, San Jose, CA, USA).

### 2.3. Identification of Strain X21196

#### 2.3.1. Morphological Identification of Strain X21196

The external morphology of the fruiting bodies and the mycelial morphology were observed. Identification was made with reference to the literature by Huang Nianlai [3] and Li Yu [12], among others.

#### 2.3.2. Molecular Identification of Strain X21196

Genomic DNA was extracted from the mycelium of this wild strain using a fungal DNA extraction kit (OMEGA, Fuzhou Nanjiang Biotechnology Co., Ltd., Fuzhou, China) following the manufacturer’s instructions. The ITS region was PCR-amplified using the primers ITS1 (5′-TCCGTAGGTGAACCTGCGG-3′) and ITS4 (5′-TCCTCCGCTTATTGATATATGC-3′). The reaction protocol followed reference: initial denaturation at 95 °C for 2 min; followed by 35 cycles of 95 °C for 15 s, 55 °C for 30 s, and 72 °C for 2 min 30 s; and a final extension at 72 °C for 5 min. A 2 μL aliquot of the PCR product was analyzed by electrophoresis on a 1% agarose gel. The purified PCR product was sent to Fuzhou Tsingke Biotechnology Co., Ltd. (Fuzhou, China) for sequencing. The obtained ITS sequence was submitted to the NCBI nucleotide database (http://www.ncbi.nlm.nih.gov, accessed on 5 August 2025) for BLAST comparison. ITS sequences with high similarity were downloaded, and a phylogenetic tree was constructed using the Maximum Likelihood method in MEGA 11.0 (Kumar Laboratory, Biodesign Institute, Arizona State University, Tempe, AZ, USA) software [21].

### 2.4. Biological Characteristics of Strain X21196

Referring to methods such as Cao et al. [22] with slight modifications. Freshly activated plate cultures were taken, and a 5 mm diameter cork borer was used to punch holes at the colony edge. The agar plugs were then inoculated onto the center of different medium plates (9 cm diameter) and incubated in the dark at 25 °C in a constant temperature incubator. After 14 days, the colony diameter was measured using the “cross” method. Each treatment was repeated five times (technical replicates).

The mycelial growth rate (mm/d) = [colony diameter (mm) − plug diameter (mm)]/growth period (d).(1)

#### 2.4.1. Effect of Carbon Source on Mycelial Growth

To investigate the effect of carbon sources on mycelial growth, a carbon source screening medium was used, with the following composition: peeled potatoes 200 g, carbon source 20 g (glucose, sucrose, fructose, maltose, mannose, or starch), peptone 5 g, MgSO_4_ 1.5 g, K_2_HPO_4_ 2 g, vitamin B1 (VB_1_) 10 mg, agar 20 g, and distilled water added to a total volume of 1 L (pH neutral).

#### 2.4.2. Effect of Nitrogen Source on Mycelial Growth

To investigate the effect of nitrogen sources on mycelial growth, a nitrogen source screening medium was used, with the following composition: peeled potatoes 200 g, glucose 20 g, nitrogen source 5 g (urea, yeast extract, ammonium sulfate, beef extract, peptone, or ammonium nitrate), MgSO_4_ 1.5 g, K_2_HPO_4_ 2 g, VB_1_ 10 mg, agar 20 g, and distilled water added to a total volume of 1 L (pH neutral).

#### 2.4.3. Effect of pH on Mycelial Growth

Enriched PDA medium was used as the pH screening medium. The pH was adjusted using 1.0 mol/L NaOH and 1.0 mol/L HCl solutions, with pH values set to 4.0, 5.0, 6.0, 7.0, 8.0, and 9.0.

#### 2.4.4. Effect of Temperature on Mycelial Growth

Enriched PDA medium was used as the temperature screening medium. After inoculation, the plates were placed in constant temperature incubators at 15, 20, 25, 30, 35, and 40 °C for incubation in darkness, with other procedures being the same.

### 2.5. Polysaccharide Extracts Antioxidant Activity Tests

#### 2.5.1. Preparation of Intracellular and Extracellular Polysaccharide Extracts

Intracellular Polysaccharide extracts (IPS): Following the method of Zhai et al. [23], the activated strain was fermented at 25 °C and 150 rpm for 10 d. The resulting mycelium was collected, freeze-dried, and ground into powder. Ten grams of the powder were mixed with 75% ethanol at a ratio of 1:20 (*w*/*v*) and subjected to ultrasonication for 2 h, after which the supernatant was discarded. The residue was mixed with water at a ratio of 1:30 (*w*/*v*) and extracted in a 90 °C water bath for 2 h; this extraction was repeated twice. The combined extracts were concentrated to one-third of their original volume. Ethanol was added to the concentrate to a final concentration of 80% for alcohol precipitation, which was carried out at 4 °C for 24 h. The precipitate was collected, redissolved, and deproteinized using the Sevag method, followed by freeze-drying. The final IPS product was stored at −20 °C.

Extracellular Polysaccharide extracts (EPS): The fermentation broth was centrifuged to obtain the supernatant. The supernatant was concentrated to one-third of its original volume, and ethanol was added to a final concentration of 80%. The mixture was left to stand at 4 °C for 24 h for precipitation. The resulting precipitate was collected, redissolved, deproteinized, and freeze-dried. The final EPS product was stored at −20 °C.

#### 2.5.2. In Vitro Antioxidant Activity Assay of Polysaccharide Extracts

##### Determination of Hydroxyl Radical (·OH) Scavenging Capacity

The method was based on Hifney et al. [24] with slight modifications. Polysaccharide extract solutions and Vc solutions were prepared at concentration gradients of 0.025, 0.05, 0.25, 0.5, 1, 2, and 5 mg/mL. Into a 96-well plate, 75 µL of polysaccharide extract solutions at different concentrations were added to respective wells, followed by sequential addition of 15 µL of FeSO_4_ solution (9 mmol/L), salicylic acid-ethanol solution (9 mmol/L), and H_2_O_2_ solution (8.8 mmol/L). The mixture was gently shaken to mix, and finally, 100 µL of distilled water was added. The plate was incubated in a constant temperature water bath at 37 °C for 30 min, after which the OD value at 510 nm was measured and recorded as A*y*. Vc was used as a positive control. A blank reaction system, where the polysaccharide extract sample was replaced with distilled water, was prepared, and its absorbance was recorded as A*o*. A control reaction system, where the H_2_O_2_ solution was replaced with distilled water, was prepared, and its absorbance was recorded as A*p*. The results were quantitatively expressed as the half-maximal inhibitory concentration (*IC*_50_ value). The *IC*_50_ value is defined as the sample concentration (mg/mL) required to scavenge 50% of the hydroxyl radicals. This value was obtained by preparing a series of sample concentration gradients, measuring their corresponding scavenging rates, and then performing non-linear regression fitting or linear fitting using IBM SPSS Statistics 27.0 software. A lower *IC*_50_ value indicates a stronger hydroxyl radical scavenging ability of the sample, i.e., higher antioxidant activity. The scavenging rate was calculated using the following formula:

Hydroxyl Radical Scavenging Rate (%) = [1 − (A*y* − A*p*)/A*o*] × 100 (2)

##### Determination of ABTS Radical Cation (ABTS·^+^) Scavenging Capacity

The method was based on Miller et al. [25] with slight adjustments. A 5 mL aliquot of 7.0 mmol/L ABTS solution was mixed with 88 µL of 2.45 mmol/L potassium persulfate aqueous solution and left to stand at room temperature in the dark for 12–16 h to prepare the ABTS·^+^ stock solution. This stock solution was diluted with distilled water, and its absorbance at 734 nm was measured using a spectrophotometer (Shimadzu, Kyoto, Japan) to achieve a value of 0.70 ± 0.02, serving as the ABTS·^+^ working solution, which was prepared fresh for each use. In a 96-well plate, 100 µL of polysaccharide extract solutions (0.025, 0.05, 0.25, 0.5, 1, 2, and 5 mg/mL) and 100 µL of the ABTS·^+^ working solution were added to respective wells, mixed thoroughly, and allowed to react in the dark at 25 °C for 20 min. The absorbance at 734 nm was measured using a microplate reader (BioTek, Winooski, VT, USA) and recorded as A*y*. Vc was used as a positive control. A blank reaction system, where the polysaccharide extract sample was replaced with distilled water, was prepared, and its absorbance was recorded as A*o*. A control reaction system, where the ABTS·^+^ working solution was replaced with distilled water, was prepared, and its absorbance was recorded as A*p*. A lower *IC*_50_ value indicates a stronger ABTS radical scavenging ability of the sample. The scavenging rate was calculated using the following formula:

ABTS Radical Scavenging Rate (%) = [1 − (A*y* − A*p*)/A*o*] × 100 (3)

##### Determination of DPPH Radical (DPPH·) Scavenging Capacity

The method was based on Saiga et al. [26] with slight modifications. In a 96-well plate, 100 µL of polysaccharide extract solutions (0.025, 0.05, 0.25, 0.5, 1, 2, and 5 mg/mL) and 100 µL of a 0.2 mmol/L DPPH solution were added sequentially to respective wells. The mixture was gently shaken to mix and allowed to react at room temperature in the dark for 30 min. The OD value at 517 nm was measured and recorded as A*y*. Vc was used as a positive control. A blank reaction system, where the polysaccharide extract sample was replaced with anhydrous ethanol, was prepared, and its absorbance was recorded as A*o*. A control reaction system, where the DPPH solution was replaced with anhydrous ethanol, was prepared, and its absorbance was recorded as A*p*. A lower *IC*_50_ value indicates a stronger DPPH free radical scavenging ability of the sample. The scavenging rate was calculated using the following formula:

DPPH Radical Scavenging Rate (%) = [1 − (A*y* − A*p*)/A*o*] × 100 (4)

##### Determination of Ferric Reducing Antioxidant Power (FRAP)

The Ferric Ion Reducing Antioxidant Power (FRAP) assay is used to evaluate the reducing ability of antioxidants. Under acidic conditions, Fe^3+^ is reduced to Fe^2+^, which reacts with TPTZ to produce a blue color. The greater the absorbance, the stronger the reducing power. The method was based on Saiga et al. [27] with slight adjustments. The FRAP working solution was prepared fresh by mixing 0.3 mol/L acetate buffer (pH 3.6), 0.02 mol/L FeCl_3_ solution, and 0.01 mol/L TPTZ solution in a volume ratio of 10:1:1.

Then, 0.5 mL of polysaccharide extract solutions (0.025, 0.05, 0.25, 0.5, 1, 2, and 5 mg/mL) were added to 3.0 mL of the FRAP working solution, mixed thoroughly, and incubated in a water bath at 37 °C for 15 min. The absorbance at 593 nm was measured and recorded as the sample absorbance (A*y*). A blank reaction system, where the polysaccharide extract sample was replaced with distilled water, was prepared, and its absorbance was recorded as (A*o*).

### 2.6. Domestication and Cultivation Experiment

#### 2.6.1. Preparation of Liquid Inoculum

Under aseptic conditions in a laminar flow hood, activated mycelial plugs were inoculated into flasks containing liquid PDA medium. The flasks were then placed in a shaking incubator at 25 °C and 160 rpm, and cultured in the dark for 9 d.

#### 2.6.2. Preparation of Cultivation Spawn and Spawn-Running Management

First, cottonseed hulls were completely submerged in water for 12 h, then thoroughly drained to prevent uneven dryness/wetness, which could lead to incomplete sterilization or excessive medium moisture hindering subsequent mycelial growth. Raw materials, including sawdust (outdoor-stacked for over three months), pre-wetted cottonseed hulls, wheat bran, lime, and white sugar, were proportionally selected. Water was added in small amounts multiple times while mixing to allow the cultivation substrate to fully absorb moisture, maintaining the water content at approximately 62%, and adjusting the pH to 5.0. The substrate was then packed into cultivation bags, with 900 g of wet substrate per bag. The bags were autoclaved at 121 °C for 2 h. After cooling to room temperature in the laminar flow hood, they were inoculated. The liquid inoculum was shaken well and carefully poured into the cultivation bags, with one flask uniformly inoculating five bags. After inoculation, the bags were transferred to a spawn-running room and incubated in the dark at 20–23 °C. During this period, the mycelial growth progress of strain X21196 and any potential contamination were monitored appropriately.

#### 2.6.3. Primordium Induction and Fruiting

Once the mycelium had fully colonized the bags and undergone a one-week maturation period, the bags were promptly moved to the fruiting room. The bags were opened to initiate fruiting. The temperature in the fruiting room was maintained at 18–20 °C, and the air humidity was increased to 95%. After primordia appeared, the temperature was controlled at 20–23 °C to allow further maturation and differentiation of the fruiting bodies.

### 2.7. Data Analysis

Data processing and analysis were performed using IBM SPSS 27.0 (IBM Corp., Armonk, NY, USA) software. Values are expressed as the mean ± standard deviation (SD). Apply the Waller-Duncan test and the *t*-test to analyze significance, where *p* < 0.05 indicates a statistically significant difference. Figures were generated using GraphPad Prism 10.0 software (GraphPad Software, LLC, Boston, MA, USA).

## 3. Results

### 3.1. Identification of Strain X21196

#### 3.1.1. Morphological Identification of Strain X21196

**Figure 1:**
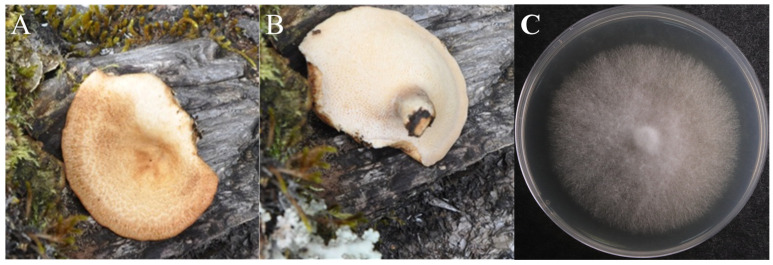
Fruit body and mycelium growth situation of strain X21196 ((**A**). Frontal side of the fruit body; (**B**). On the opposite side of the fruit body; (**C**). Colony morphology).

The morphology of the wild X21196 fruiting body is shown in Figure 1A,B. The fruiting body grows in broad-leaved forests, is annual. The pileus is semicircular, concave in the center. The surface is yellowish/brown to ochre, covered with tea-brown or dark brown patches, thinning from the base towards the margin. The underside of the fruiting body bears dense pores, with the pore surface pale yellowish/brown. The stipe is lateral and covered with tomentum. The context is white to creamy, fleshy. The mycelium appears white on PDA enrichment medium, with abundant aerial hyphae radiating from the center outwards (Figure 1C). The above characteristics correspond with those of *P. tuberaster* described in the Illustrated handbook of macrofungal resources in China [12]. Therefore, strain X21196 is preliminarily identified as *P. tuberaster*.

#### 3.1.2. Molecular Identification of Strain X21196

**Figure 2:**
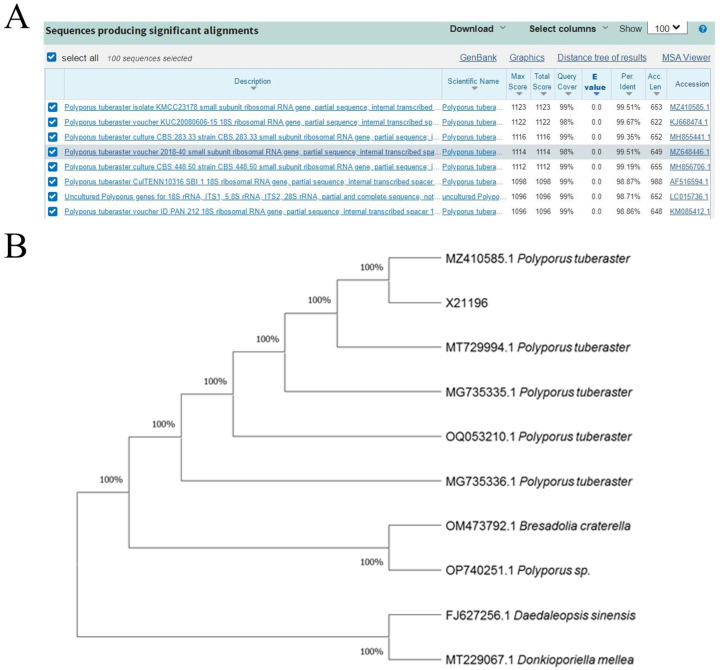
Molecular Identification of Strain X21196 ((**A**). Blast alignment results of strain X21196 ITS sequence in NCBI database; (**B**). Phylogenetic tree constructed based on ITS fragment maximum likelihood method).

The ITS sequence of strain X21196 is 620 bp in length. A BLAST comparison against the NCBI database revealed that it shares the highest similarity (99.51%) with the ITS sequence of *P. tuberaster* (MZ410585.1), as shown in Figure 2A. A phylogenetic tree was constructed using other ITS sequences that showed high similarity with the strain’s sequence (Figure 2B). The results indicated that this strain clusters within the same clade as *Polyporus tuberaster*, confirming that they belong to the same species. Therefore, based on the combined morphological and molecular analyses, the strain is conclusively identified as *P. tuberaster*. The ITS sequence of strain X21196 is openly available in the NCBI accession number: PQ094097.1.

### 3.2. Study on Biological Characteristics of Strain X21196

#### 3.2.1. Effect of Different Carbon Sources on the Mycelial Growth of *P*. *tuberaster* X21196

**Figure 3:**
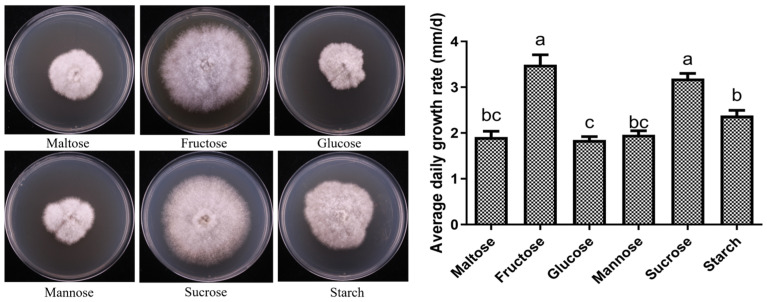
Effect of carbon source on the mycelial growth of *P. tuberaster* X21196 (IMaltose, Fructose, Glucose, Mannose, Sucrose, Starch). On the bar chart, within each group, the same letters indicate no significant difference, while different letters denote significant differences (*p* < 0.05). The same convention applies below.

The mycelial growth of X21196 under different carbon source conditions is shown in Figure 3. The mycelium of *P. tuberaster* X21196 was able to grow under all tested carbon source conditions, but the growth performance varied. When fructose was used as the carbon source, the mycelium exhibited the fastest growth rate, was dense, and had a regular margin; sucrose was the next best. In contrast, when starch, maltose, mannose, and glucose served as carbon sources, the mycelial growth rate was slower, and the margins were irregular. Based on the analysis of mycelial growth speed and morphology, the preference of *P. tuberaster* X21196 for the six tested carbon sources is as follows: fructose (3.492 mm/d) > sucrose (3.189 mm/d) > starch (2.383 mm/d) > mannose (1.961 mm/d) > maltose (1.911 mm/d) > glucose (1.850 mm/d). Therefore, fructose is the preferred carbon source for *P. tuberaster* X21196.

#### 3.2.2. Effect of Different Nitrogen Sources on the Mycelial Growth of *P*. *tuberaster* X21196

**Figure 4:**
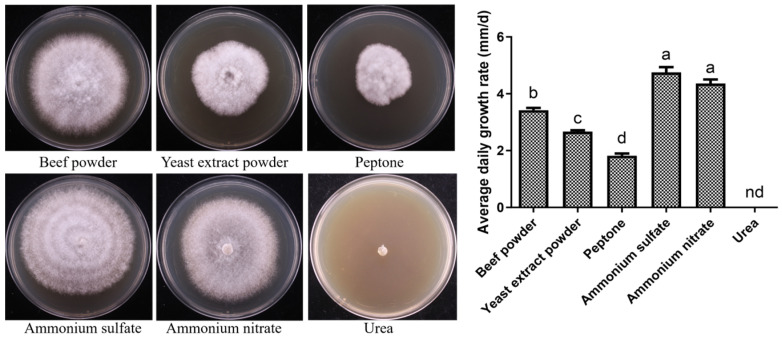
Effect of nitrogen source on the mycelial growth of *P. tuberaster* X21196 (Beef powder, Yeast extract powder, Peptone, Ammonium sulfate, Ammonium nitrate, VI: Urea). nd: not detected. On the bar chart, within each group, the same letters indicate no significant difference, while different letters denote significant differences (*p* < 0.05).

The mycelial growth of X21196 under different nitrogen source conditions is shown in Figure 4. The mycelium of *P. tuberaster* X21196 was capable of growth under most of the tested nitrogen sources, but the growth performance varied. When ammonium sulfate was used as the nitrogen source, the mycelium exhibited the fastest growth rate and was dense, followed by ammonium nitrate. In contrast, when beef extract, yeast extract, and peptone served as nitrogen sources, the mycelial growth rate was slower, and no growth occurred with urea as the nitrogen source. Based on the analysis of mycelial growth speed and morphology, the preference of *P. tuberaster* X21196 for the six tested nitrogen sources is as follows: ammonium sulfate (4.750 mm/d) > ammonium nitrate (4.356 mm/d) > beef extract (3.422 mm/d) > yeast extract (2.667 mm/d) > peptone (1.822 mm/d) > urea (0 mm/d). Therefore, ammonium sulfate is the preferred nitrogen source for *P. tuberaster* X21196.

#### 3.2.3. Effect of Different Temperatures on the Mycelial Growth of *P. tuberaster* X21196

**Figure 5:**
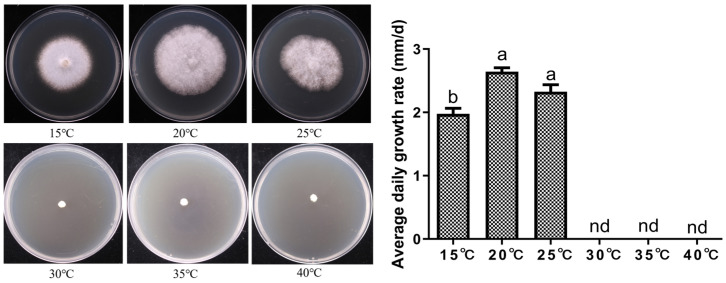
Effect of temperature on the mycelial growth of *P. tuberaster* X21196 (I: T = 15 °C, II: T = 20 °C, III: T = 25 °C, IV: T = 30 °C, V: T = 35 °C, VI: T = 40 °C). nd: not detected. On the bar chart, within each group, the same letters indicate no significant difference, while different letters denote significant differences (*p* < 0.05).

The mycelial growth of *P. tuberaster* X21196 under different temperature conditions is shown in Figure 5. The tested temperatures had a significant impact on mycelial growth. Based on the growth status of the mycelium, it is evident that *P. tuberaster* X21196 does not favor high temperatures. It grew relatively well within the range of 15–25 °C, with 20–25 °C being the optimal temperature range. No mycelial growth was observed when the temperature exceeded 30 °C.

#### 3.2.4. Effect of Different pH Levels on the Mycelial Growth of *P*. *tuberaster* X21196

**Figure 6:**
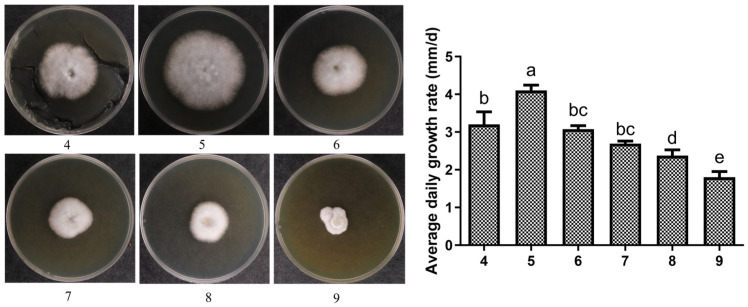
Effect of pH on the mycelial growth of *P. tuberaster* X21196 (I: pH = 4.0, II: pH = 5.0, III: pH = 6.0, IV: pH = 7.0, V: pH = 8.0, VI: pH = 9.0). nd: not detected. On the bar chart, within each group, the same letters indicate no significant difference, while different letters denote significant differences (*p* < 0.05).

The mycelial growth of *P. tuberaster* X21196 under different pH conditions is shown in Figure 6. It was capable of growth within the pH range of 4–9, but the growth performance varied. At pH 4.0, the solidification ability of the medium was relatively poor, yet the mycelium grew normally. The mycelial growth rate was fastest at pH 5.0, with neat colony margins and dense mycelia. Beyond pH 5.0, as the pH value increased, the mycelial growth rate gradually decreased.

### 3.3. Chemical Antioxidant Activity of Intracellular and Extracellular Polysaccharide Extracts

#### 3.3.1. Scavenging Capacity of Polysaccharides on ABTS Free Radicals

**Figure 7:**
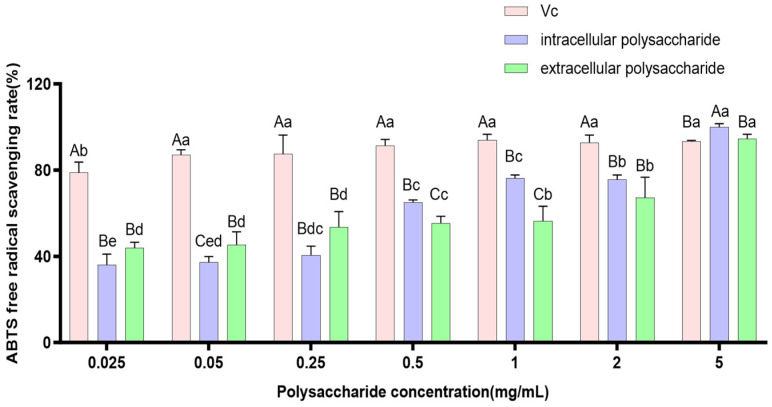
Scavenging capacity of *P*. *tuberaster* polysaccharide extracts on ABTS free radicals. On the bar chart, the same letters indicate no significant difference, while different letters denote a significant difference (*p* < 0.05). Uppercase letters represent differences between groups, and lowercase letters represent differences within groups. The same convention applies to the following figures.

ABTS is a water-soluble free radical initiator that, under the action of an oxidant, is oxidized to green ABTS·^+^, which exhibits maximum absorption at 734 nm. In the presence of antioxidant substances, ABTS·^+^ is reduced, resulting in color fading and a decrease in absorbance. The change in absorbance can be used to evaluate the antioxidant activity of compounds. This method is rapid, easy to operate, and widely used to assess the total antioxidant capacity of compounds [28]. The ABTS·^+^ radical scavenging ability of *P. tuberaster* polysaccharide extracts is shown in Figure 7. Both polysaccharide extracts exhibit good scavenging activity against ABTS·^+^ radicals, and the scavenging effect increases with increasing mass concentration. By comparing the calculated *IC*_50_ values for extracellular polysaccharide extracts (0.125 ± 0.048 mg/mL) and intracellular polysaccharide extracts (0.152 ± 0.027 mg/mL). Although the extracellular polysaccharide extracts of *P. tuberaster* exhibited higher ABTS radical scavenging capacity than the intracellular polysaccharide extracts, the difference did not reach statistical significance.

#### 3.3.2. Scavenging Ability of Polysaccharides on DPPH Free Radicals

**Figure 8:**
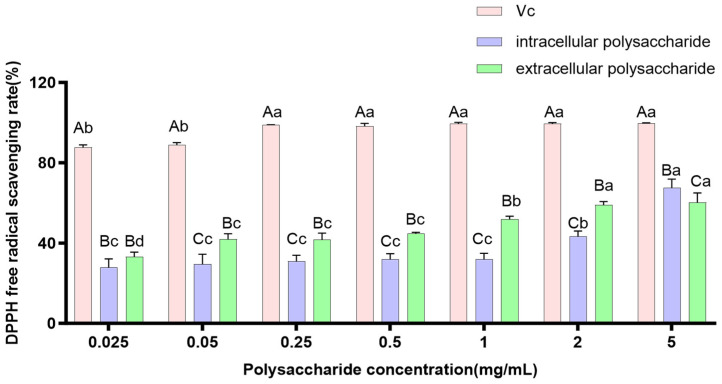
Scavenging capacity of *P. tuberaster* polysaccharide extracts on DPPH free radicals. Uppercase letters represent differences between groups, and lowercase letters represent differences within groups.

As a model compound for lipid-free radicals, the DPPH free radical serves as a crucial indicator for evaluating the free radical scavenging activity of compounds [29]. The scavenging effects of intracellular and extracellular polysaccharide extracts from *P. tuberaster* fungus on DPPH free radicals were relatively similar. Within the concentration range of 0.025–5.000 mg/mL, the intracellular and extracellular polysaccharide extracts of *P. tuberaster* exhibited similar trends in scavenging DPPH free radicals, with their scavenging capacity increasing as the concentration increased (Figure 8). The IC_50_ values for the extracellular and intracellular polysaccharide extracts were calculated to be 0.683 ± 0.116 mg/mL and 3.401 ± 0.619 mg/mL, respectively. By comparing the *IC*_50_ values, it was found that the extracellular polysaccharide extracts possessed a stronger DPPH free radical scavenging ability than the intracellular polysaccharide extracts (*p* < 0.05).

#### 3.3.3. Scavenging Ability of Polysaccharides on ·OH Free Radicals

**Figure 9:**
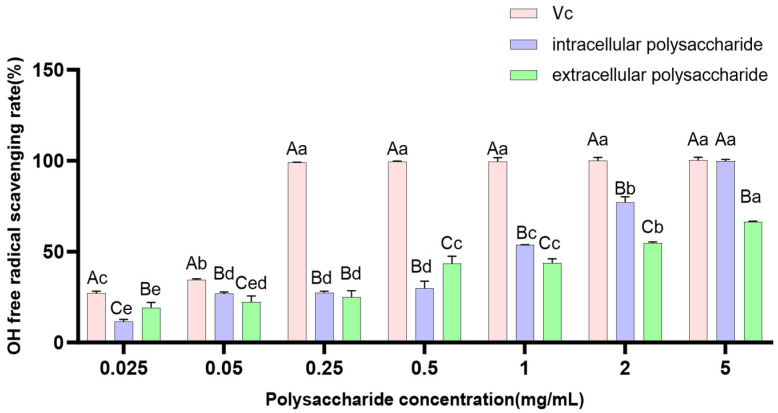
Scavenging capacity of *P. tuberaster* polysaccharides extracts on ·OH free radicals. Uppercase letters represent differences between groups, and lowercase letters represent differences within groups.

The hydroxyl radical (·OH) is a highly reactive and aggressive free radical, widely recognized as the most toxic reactive oxygen species. It poses strong, harmful effects on organisms and is a major factor causing peroxidative damage in living systems [30]. The ability of an antioxidant to scavenge ·OH radicals is often used as an indicator to evaluate its antioxidant activity. Within the concentration range of 0.025–5.00 mg/mL, the ·OH radical scavenging assay (Figure 9) showed that both extracellular and intracellular polysaccharide extracts from *P. tuberaster* fungus exhibited certain scavenging effects on ·OH radicals, which were positively correlated with concentration. By fitting the equations, the *IC*_50_ values for ·OH radical scavenging were calculated to be 1.357 ± 0.198 mg/mL and 0.595 ± 0.046 mg/mL for the extracellular and intracellular polysaccharide extracts, respectively. This indicates that, within the tested concentration range, the intracellular polysaccharide extracts of *P. tuberaster* fungus possess higher ·OH radical scavenging activity than the extracellular polysaccharide extracts (*p* < 0.01).

#### 3.3.4. Ferric Ion Reducing Power (FRAP) of Polysaccharides

The FRAP method is based on the reduction in Fe^3+^ to Fe^2+^, which then reacts with TPTZ to form a blue complex. The stronger the reducing power, the higher the antioxidant activity, and the deeper the blue color of the reaction system [31]. Within the concentration range of 0.025–5.000 mg/mL, both polysaccharide extracts exhibited certain reducing capabilities (Table 1). As the polysaccharide extract concentration increased, the reducing power gradually enhanced in a dose-dependent manner. The intracellular polysaccharide extracts showed a stronger reducing power than the extracellular polysaccharide. At the polysaccharide extracts concentration of 5 mg/mL, the FRAP values reached their highest, which were 0.1708 and 0.1582, respectively.

### 3.4. Mycelial Running, Primordium Induction, and Fruiting

After 5 d of liquid culture, the inoculum block of *P. tuberaster* strain X21196 formed a white, spiny spherical shape, and numerous millet-sized mycelial pellets appeared in the liquid medium. By day 9, a large number of mycelial pellets were evenly distributed throughout the liquid medium, marking the end of the liquid culture phase. The liquid inoculum was immediately transferred to cultivation bags, with approximately 20 mL per bag, and incubated at a constant temperature of 23 °C in the dark, maintaining 65% air humidity. After 32 d, the mycelium fully colonized the bags, followed by a 7 d maturation period. Under suitable conditions, pale yellow primordia appeared after 12 d. The temperature was maintained at 20–23 °C with air humidity above 90%. The fruiting bodies were harvested 36 d after bag opening when they reached maturity, indicated by the clear formation of pores.

### 3.5. Agronomic Traits of Fruiting Bodies

**Figure 10:**
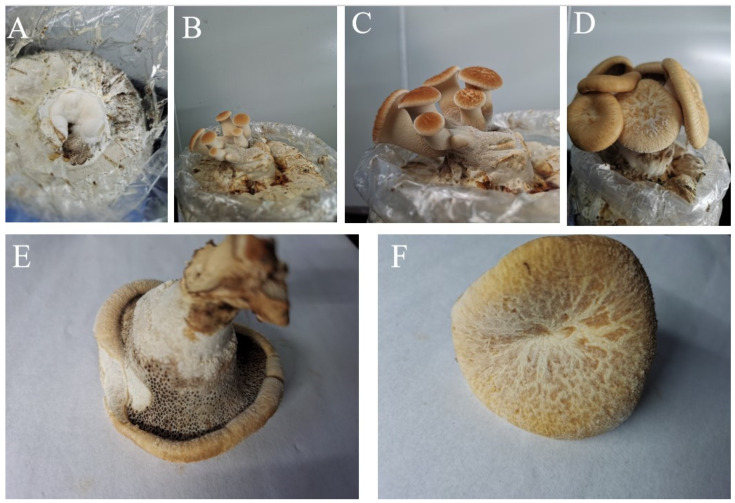
Growth situation of artificially domesticated fruiting bodies of *P*. *tuberaster* ((**A**–**C**) refer to the tender stage of artificially cultivated fruiting bodies; (**D**) is the mature stage of the cultivated fruiting body; (**E**) represents the mature artificial cultivated fruiting body on the front side; (**F**) represents the opposite side of the mature artificially cultivated fruiting body).

Each cultivation bag had a dry substrate weight of 900 g. The average fresh weight of the first flush of fruiting bodies per bag was 41.27 g. The growth of cultivated fruiting bodies is shown in Figure 10. As illustrated, the mycelium of *P. tuberaster* X21196 is white, and the primordia are initially pale yellow (Figure 10A). As the fruiting bodies develop, they gradually turn yellowish/brown (Figure 10B–D). The pileus is circular with a central depression, surface colored yellowish/brown to ochre, and covered with tea-brown or dark brown patches, thinning from the base toward the margin. The lower surface of the fruiting body bears dense pores, and the stipe is lateral and covered with fine hairs. The context is white to creamy and fleshy in texture (Figure 10E,F). These morphological characteristics are consistent with those of wild fruiting bodies.

## 4. Discussion

Traditional fungal classification is based on the observation and description of morphological characteristics. Researchers primarily categorize fungi into different groups by examining features such as hyphal morphology, spore shape, cap and gill structures under a microscope [32]. However, fruiting bodies are susceptible to polymorphism due to factors like growth environment and nutritional conditions, which to some extent limits the development of taxonomy. Nevertheless, with the advancement of molecular biology techniques, molecular taxonomy has emerged. Molecular taxonomy utilizes differences in fungal DNA sequences to determine phylogenetic relationships and evolutionary distances among species [33]. Among these, the ITS rDNA region evolves rapidly, exhibits extensive sequence polymorphism, is relatively short, and is easy to amplify and sequence, making it widely used in fungal classification [1]. By combining morphological characteristics with molecular evidence, more accurate identification and classification of fungi can be achieved. In this study, a wild polypore fruiting body was isolated and identified through a combination of classical morphology and molecular biology. It was found that the ITS sequence of strain X21196 is most closely related to that of *P*. *tuberaster*, with a similarity of 99.51%. Moreover, this strain shares multiple morphological characteristics with *P*. *tuberaster*, confirming it as *P*. *tuberaster*.

Subsequently, studies on biological characteristics revealed that experiments on the effects of different carbon and nitrogen sources on the mycelial growth of *P*. *tuberaster* X21196 showed that the mycelium could grow on all tested carbon source media, with fructose being the optimal carbon source. This aligns with the biological characteristics of *Ganoderma gibbosum* [34] and *Trametes strumosa* [35]. The growth of mycelium varied across different nitrogen source media. With inorganic nitrogen sources such as ammonium nitrate and ammonium sulfate, the mycelium grew rapidly, was pure white and dense, and the colony edges were neat, with ammonium sulfate being the optimal nitrogen source. However, with organic nitrogen sources such as beef extract, yeast extract, peptone, and urea, the mycelial growth was slower, the colony edges were irregular, and in some cases, normal growth was inhibited. This indicates that *P*. *tuberaster* has a preference for inorganic nitrogen sources. The reason may be that inorganic nitrogen sources have high solubility and are easily broken down into various free inorganic ions. Additionally, the strain possesses its own unique enzyme system, allowing it to directly absorb inorganic nitrogen sources to synthesize proteins and other essential biological molecules [36]; hence, it grows better in the presence of inorganic nitrogen sources. In temperature experiments, *P*. *tuberaster* X21196 could grow at temperatures between 15 and 25 °C, with no significant difference in growth speed at 20 °C and 25 °C, making 20–25 °C the optimal temperature range. Above 25 °C, the growth rate decreased, and growth ceased beyond 30 °C, indicating that this strain is a low-temperature fungus. This result is consistent with the climate at the collection time and location. Orthogonal experiments showed that the optimal temperature for *P*. *tuberaster* X21196 is 25 °C, which aligns with the findings of Cheng Guohui et al. [13]. In single-factor pH experiments, the mycelium of *P*. *tuberaster* X21196 could grow at pH values ranging from 4.0 to 9.0, with an optimal pH of 5.0. Beyond pH 5.0, the growth rate decreased as pH increased. This result is consistent with observations for other polypores such as *Trametes velutina* [37] and *Polyporus arcularius* [38]. These findings provide important references for subsequent liquid spawn preparation, cultivation bag culture, and fruiting body management.

Free radicals are ubiquitous in living organisms. Under normal physiological conditions, their production and clearance are in a dynamic equilibrium, and physiological concentrations of free radicals are necessary for metabolism [39]. When the body suffers from oxidative damage, excessive free radicals can cause oxidative damage to tissues and cells, leading to aging. Studies have also shown that an imbalance in free radical levels can induce various diseases, such as tumors [40,41]. To prevent or mitigate the harm caused by free radicals, appropriate exogenous antioxidants are needed to enhance the body’s antioxidant capacity. Since most chemically synthesized antioxidants have toxic side effects, researching and developing natural antioxidants to replace synthetic ones has become a hot topic. For instance, Wang et al. isolated and purified a novel homogeneous polysaccharide (HVP-1) with antioxidant activity from *Volvariella volvacea* [42], while Petraglia Tania et al. isolated a polysaccharide-enriched substance from the edible mushroom *Pleurotus eryngii*, which also exhibited antioxidant properties [43]. In this study, the extracellular and intracellular polysaccharide extracts from the mycelium of *P*. *tuberaster* were tested for their in vitro antioxidant capacity. The results showed that both polysaccharide extracts possess certain antioxidant activities, but their performances differed. The extracellular polysaccharide extracts exhibited stronger scavenging ability against ABTS and DPPH free radicals compared to the intracellular polysaccharide extracts, while the intracellular polysaccharide extracts showed stronger scavenging ability against OH free radicals and Fe^3+^ reducing capacity. Additionally, the scavenging ability increased with higher concentrations, which may be due to differences in the specificity and sensitivity of the samples to these four indicators [44].

Although in vitro experiments have confirmed the antioxidant potential of polysaccharide extracts, their in vivo application still requires validation due to the more complex mechanisms of polysaccharides in biological systems. The in vivo antioxidant mechanisms of polysaccharides are closely related to their structural characteristics. Edible fungal polysaccharides are primarily classified into α-glucans and β-glucans, which follow different pathways in the digestive tract [45]. α-Glucans (such as starch-like polysaccharides) can be partially degraded by mammalian digestive enzymes (e.g., α-amylase) in the small intestine, breaking down into oligosaccharides or monosaccharides before absorption [46]. In contrast, β-glucans, particularly those with a β-(1→3) main chain and β-(1→6) branched structure characteristic of fungal polysaccharides, resist direct enzymatic digestion in the stomach and small intestine due to the absence of corresponding hydrolytic enzymes in mammals. Most of them reach the colon intact [45,46]. Once in the colon, β-glucans can serve as prebiotics, fermented by the gut microbiota to produce short-chain fatty acids (SCFAs). These SCFAs not only provide energy for intestinal epithelial cells but also enhance intestinal barrier function by activating the AMPK signaling pathway and inhibiting NF-κB activation, thereby reducing oxidative stress and inflammatory responses [47,48]. Furthermore, β-glucans can be recognized by pattern recognition receptors (such as Dectin-1) on the surface of intestinal immune cells, modulating immune responses and consequently influencing systemic redox status. They may even enter Peyer’s patches via transcytosis through intestinal M cells, initiating mucosal immune responses [49,50,51]. Additionally, the interaction between polysaccharides and the colonic mucus barrier affects mucin secretion and degradation, mucus viscosity and structure, barrier integrity, and epithelial and immune signaling, particularly in protecting against oxidative stress-induced mucosal dysfunction [52]. However, this study only evaluated the in vitro activity of the polysaccharide extract, and the sample used was a crude extract. Therefore, it may contain non-polysaccharide components such as polyphenols and proteins, which may contribute to the observed antioxidant activity and no in vivo experiments have been conducted for validation. Future research should focus on the purification and structural characterization of the polysaccharide extract, combined with animal models and gut microbiota analysis, to further elucidate its in vivo mechanism of action and structure-activity relationship. This will provide a theoretical basis for the development of natural antioxidants and functional foods derived from *P. tuberaster* sclerotia.

Currently, there are few reports on the cultivation of *P*. *tuberaster*. This study successfully achieved its artificial domestication and cultivation. Under conditions of 23 °C, 160 r/min, and dark culture, liquid spawn was prepared in 9 d. At 23 °C and dark culture, mycelium fully colonized the cultivation bags in 32 d. With air humidity above 95% and appropriate scattered light stimulation, pale yellow primordia formed after 12 d. Subsequently, under conditions of 85–90% air humidity and temperatures of 20–23 °C, the fruiting bodies matured after 24 d. Compared to the traditional three-tier spawn system used by Cheng et al. [13], this cultivation time was significantly shortened. The primordia were initially pale yellow, gradually turning yellowish/brown as the fruiting bodies grew. The caps were circular, centrally depressed, with surfaces ranging from yellowish/brown to ochre, covered with tea-brown or dark brown patches, thinning from the base to the edges. The undersurface of the fruiting bodies had dense pores, and the stipe was lateral and covered with fine hairs. The flesh was white to creamy and fleshy, consistent with the morphological characteristics of wild fruiting bodies. Cultivation bags were automatically filled and sealed by machines, each with a constant dry weight of 900 g. The average fresh yield per bag for the first flush was 41.27 g. The low yield per fruiting body may be related to the cultivation substrate formula and management methods, suggesting the need for optimization in the future to improve yield.

The results of this study provide a research foundation for the conservation of *P*. *tuberaster* resources and the development and utilization of its extract products.

## Figures and Tables

**Table 1:** Ferric ion reducing antioxidant power (FRAP) of *P. tuberaster*.

Concentration (mg/mL)	Vc	Intracellular Polysaccharide	Extracellular Polysaccharide
0.0250	0.4132 ± 0.0278 Ac	0.0142 ± 0.0052 Bd	0.0072 ± 0.0043 Bc
0.0500	0.6630 ± 0.0247 Ab	0.0801 ± 0.0070 Bc	0.0128 ± 0.0055 Cc
0.2500	0.7846 ± 0.0547 Aa	0.0719 ± 0.0123 Bc	0.0141 ± 0.0094 Bc
0.5000	0.7637 ± 0.0192 Aa	0.0792 ± 0.0151 Bc	0.0174 ± 0.0103 Cc
1.0000	0.7637 ± 0.0440 Aa	0.0752 ± 0.0049 Bc	0.0216 ± 0.0130 Cc
2.0000	0.7656 ± 0.0480 Aa	0.1412 ± 0.0178 Bb	0.1147 ± 0.0115 Bb
5.0000	0.7316 ± 0.0159 Aa	0.1708 ± 0.0171 Ba	0.1582 ± 0.0078 Ba

Uppercase letters represent differences between groups, and lowercase letters represent differences within groups.

## Data Availability

The ITS sequence of strain X21196 is openly available in the NCBI accession number: PQ094097.1. The original contributions presented in the study are included in the article further inquiries can be directed to the corresponding author.
